# Randomized Adjuvant Chemotherapy of *EGFR*-Mutated Non-Small Cell Lung Cancer Patients with or without Icotinib Consolidation Therapy

**DOI:** 10.1371/journal.pone.0140794

**Published:** 2015-10-16

**Authors:** Siyang Feng, Yuanyuan Wang, Kaican Cai, Hua Wu, Gang Xiong, Haofei Wang, Ziliang Zhang

**Affiliations:** 1 Department of Thoracic Surgery, Nanfang Hospital, Southern Medical University, Guangzhou, Guangdong, China; 2 Department of Oncology, Nanfang Hospital, Southern Medical University, Guangzhou, Guangdong, China; Catalan Institute of Oncology, SPAIN

## Abstract

**Background:**

Epidermal growth factor receptor (*EGFR*) mutations occur in up to 50% of Asian patients with non-small cell lung cancer (NSCLC). Treatment of advanced NSCLC patients with *EGFR*-tyrosine kinase inhibitor (*EGFR*-TKI) confers a significant survival benefit. This study assessed the efficacy and safety of chemotherapy with or without icotinib in patients undergoing resection of stage IB to ⅢA *EGFR*-mutated NSCLC.

**Methods:**

Patients with surgically resected stage IB (with high risk factors) to ⅢA *EGFR*-mutated NSCLC were randomly assigned (1:1) to one of two treatment plans. One group received four cycles of platinum-based doublet chemotherapy every three weeks, and the other group received platinum-based chemotherapy supplemented with consolidation therapy of orally administered icotinib (125 mg thrice daily) two weeks after chemotherapy. The icotinib treatment continued for four to eight months, or until the occurrence of disease relapse, metastasis or unacceptable icotinib or chemotherapy toxicity. The primary endpoint was disease-free survival (DFS).

**Results:**

41 patients were enrolled between Feb 9, 2011 and Dec 17, 2012. 21 patients were assigned to the combined chemotherapy plus icotinib treatment group, while 20 patients received chemotherapy only. DFS at 12 months was 100% for icotinib-treated patients and 88.9% for chemotherapy-only patients (p = 0. 122). At 18 months DFS for icotinib-treated vs. chemotherapy-only patients was 95.2% vs. 83.3% (p = 0. 225), respectively, and at 24 months DFS was 90.5% vs. 66.7% (p = 0. 066). The adverse chemotherapy effects predominantly presented as gastrointestinal reactions and marrow suppression, and there was no significant difference between the two treatment groups. Patients in the chemotherapy plus icotinib treatment group showed favorable tolerance to oral icotinib.

**Conclusions:**

The results suggest that chemotherapy plus orally icotinib displayed better DFS compared with chemotherapy only, yet the difference in DFS was not significant. We would think the preliminary result here was promising, and further trials with larger sample sizes might confirm the efficiency of adjuvant TKI in selected patients.

**Trial Registration:**

ClinicalTrials.gov NCT02430974

## Introduction

Lung cancer is the leading cause of cancer-related mortality worldwide and non-small cell lung cancer (NSCLC) accounts for more than 85% of all lung cancer cases [1]. Although stage I~ⅢA NSCLC can be radically resected, the overall five-year survival rate of these patients is limited to 23~67% [2]. Patients with stage IB (with high risk factors) to stage ⅢA are recommended to receive postoperative adjuvant chemotherapy for better survival, but they are relatively insensitive to such treatment [3–10].

Epidermal growth factor receptor (*EGFR*) overexpression or over-activity occurs frequently in NSCLC patients [11, 12]. *EGFR*-tyrosine kinase inhibitors (*EGFR*-TKIs) can competitively inhibit the ATPs binding to the intracellular areas of *EGFR* and block its signaling pathway, thus achieving the anti-tumor effect [13–15]. *EGFR*-TKIs are promising therapeutic drugs for the effective in treatment of *EGFR*-mutated NSCLC because patients with *EGFR* activating mutations are notably sensitive to *EGFR*-TKIs [16, 17]. There are four drug-sensitive mutations, including point mutations in exon 18 (G719A/C), 21 (L858R and L861Q) and in-frame deletions in exon 19[18]. These sensitizing *EGFR* mutations are found in approximately 10% of Caucasian patients with NSCLC and up to 50% of Asian patients [19].

A number of clinical trials have demonstrated that there is a significant survival benefit associated with TKI treatment in patients with *EGFR*-mutated advanced NSCLC [20–27]. Other combined therapeutic regimens of chemotherapy and TKIs, such as sequential therapy of chemotherapy followed by TKI [28–30] and intercalated combination of chemotherapy and TKI [31, 32], significantly improved progression-free survival (PFS) in advanced NSCLC.

In this study, we hypothesized that targeted TKI treatment could be used as an effective consolidation therapy to enhance postoperative adjuvant therapy after regular radical surgery and adjuvant chemotherapy. Icotinib, an orally administered TKI, is an approved therapeutic for the treatment of advanced NSCLC. In a previous phase 3 study (ICOGEN), icotinib showed similar efficacy and a better safety profile when compared to gefitinb [33]. Therefore, we assessed the efficacy and safety of chemotherapy with or without orally administered icotinib treatment for patients undergoing resection of stage IB (with high risk factors) to ⅢA *EGFR*-mutated NSCLC.

## Methods

### Population

This study was undertaken at Nanfang Hospital, Southern Medical University (Guangzhou, China). The clinical stage of the patient with a lung lesion was assessed by the following: 1) Positron emission tomography-computed tomography (PET-CT) or 2) Enhanced chest X-ray computed tomography (CT) scan, brain magnetic resonance imaging (MRI), bone scan and abdominal ultrasound, whether a bronchoscopy need to take was depended. Patients would receive unidirectionally thoracoscopic lobectomy and lymphadenectomy [34, 35] if the lung lesion was considered to be removed completely in surgery. Tumor specimens were collected during surgery and used for pathology diagnosis to confirm the exact pathology classification, tumor differentiation and the pTNM stage. *EGFR* gene mutation was detected by the scorpion amplification refractory mutation system (ARMS method).

Patients were considered eligible for study inclusion if they were over 18 years of age, received an operation to remove the lung lesion completely, and had histologically confirmed activating *EGFR*-mutated NSCLC between stage IB (with high risk factors) and stage ⅢA, an Eastern Cooperative Oncology Group (ECOG) performance status of 0 or 1, adequate hematological, biochemical and organ function. Patients with high-risk stage IB NSCLC were defined as those with poorly differentiated tumors (including lung neuroendocrine tumors, but excluding well-differentiated neuroendocrine tumors), vascular invasion, wedge resection, tumor size > 4 cm, visceral pleural involvement or incomplete lymph node sampling. Patients were judged to have activating *EGFR*-mutation-positive disease if one or more of four mutations (exon 19 deletion, or 18 G719X, 21 L858R, or 21 L861Q mutations) [18] were detected.

Those with a single mutation of exon 20 T790M, 20 insertions or 19 D761Y [18] were considered to be resistant to *EGFR*-TKI and were excluded from this study. Other exclusion criteria included systemic anticancer therapy prior to surgery, other malignancies before or during the study, any unstable illness, pregnancy or lactation.

This study was approved by the Medical Ethics Committee of Nanfang Hospital and performed in accordance with the Declaration of Helsinki and Good Clinical Practice guidelines. All patients provided written informed consent before participating in this study.

### Study design

Eligible patients were randomly assigned in a 1:1 ratio to the chemotherapy-only or chemotherapy plus icotinib treatment group by using a random digit table. All patients received four cycles of platinum-based doublet chemotherapy (150 mg/m^2^ paclitaxel plus 80 mg/m^2^ nedaplatin or 30mg/m^2^ lobaplatin on day one of a three-week cycle). Two weeks after chemotherapy finishing, patients assigned to the consolidation therapy group began oral icotinib treatment (125 mg, thrice daily). Icotinib treatment continued for four to eight months, or until the occurrence of disease relapse, metastasis or unacceptable icotinib or chemotherapy toxicity.

The treatment response was assessed by the following: 1) PET-CT or 2) Enhanced chest CT scan, brain MRI, bone scan and abdominal ultrasound at the beginning of the fourth chemotherapy cycle and every six months thereafter. The primary endpoint of this study was disease-free survival (DFS), which was defined as the time from surgery to the first confirmed occurrence of disease relapse or metastasis. The secondary endpoint evaluated the acceptable toxicity of chemotherapy and oral icotinib treatment. Toxicity was classified according to the World Health Organization Toxicity Grading Scale for Determining the Severity of Adverse Events. Those patients will be considered as not completed the planned treatment program who received less than 4 cycles of chemotherapy and (or) less than 4 months of oral icotinib.

### Statistical analysis

We calculated a sample size of 26 patients per group, assuming a type I error of 0.05 (two-sided), an 80% power of the test, a two-year DFS of 60% for chemotherapy [8], an assumed two-year DFS of 95% for chemotherapy plus icotinib consolidation therapy, a 1:1 ratio of the sample sizes of the two groups. Since the anticipated dropout rate was 10%, the optimum sample size would be 29 patients per group in this study. The sample size calculation was performed using PASS 11.0 statistical software (NCSS LLC., Kaysville, UT, USA).

The full analysis set comprised all randomly assigned patients, but rejecting those mistakenly enrolled, or did not receive any allocated treatment or follow-up. The per-protocol set comprised patients who finished the planned treatment program. The safety set comprised patients who receive at least one dose of allocated treatment. All statistical analyses were performed using SPSS 13.0 statistical software (SPSS Inc., Chicago, IL, USA). Kaplan-Meier curves were used to describe survival data, and a two-sided log-rank test was used to compare the two treatment groups. The cutoff for the primary analysis was 24 months after the last patient randomly assigned. Clinical measurements were analyzed with the Student’s *t*-test, unordered enumeration data with the χ^2^ test and ranked data with the Wilcoxon rank sum test. A *p* ≤ 0.05 was considered statistically significant.

This trial is registered with ClinicalTrials.gov, number NCT02430974. The registration was completed after the enrollment of participants started, which might be largely due to the lack of enough understanding to the registration policies.

## Results

### Patient characteristics

**Fig 1:**
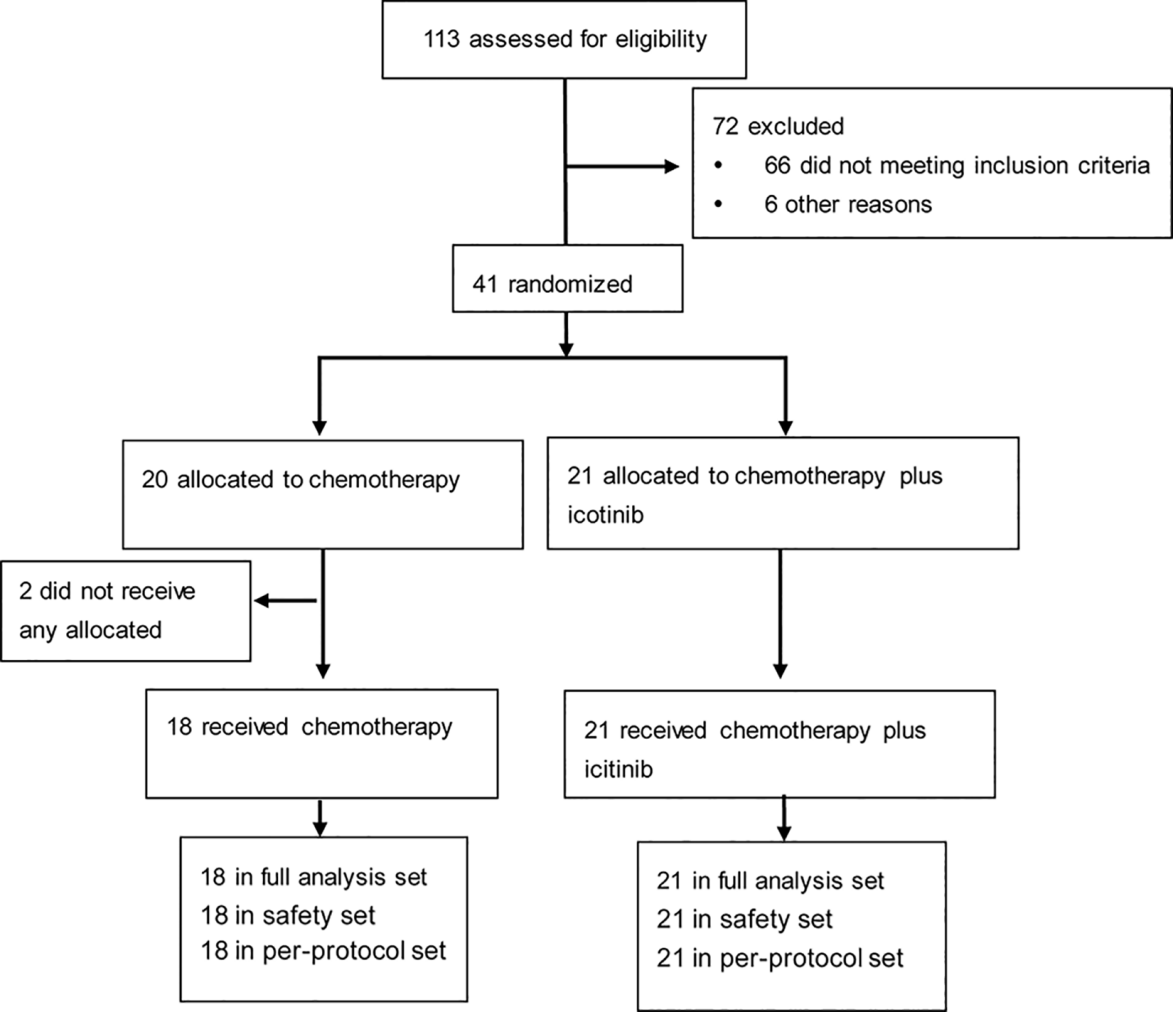
Trial profile.

113 patients were assessed for the inclusion criteria between Feb 9, 2011 and Dec 17, 2012, 72 were excluded mostly for *EGFR* wild-type NSCLC. 41 patients were randomly assigned to the chemotherapy-only (n = 20) or chemotherapy plus icotinib (n = 21) treatment group. Two patients in the chemotherapy group did not receive any study treatment after random assignment and were removed from the analysis. The data cutoff for the primary analysis was Dec 30, 2014. There were 18 patients in the chemotherapy-only group and 21 patients in the chemotherapy plus icotinib group (Fig 1). Among the enrolled patients, 17 (43.6%) had high-risk stage IB, 10 (25.6%) had stageⅡ, and 12 (30.8%) had stage ⅢA NSCLC. Because the sample size is smaller than 40 cases, the Fisher's exact probability method was used to analyze unordered enumeration data instead of the scheduled χ^2^ test. Baseline demographics and disease characteristics were balanced between the two treatment groups (Table 1).

**Table 1:** Patient Characteristics.

	Chemotherapy	Chemotherapy plus icotinib
	(n = 18)	(n = 21)
	n (%)	n (%)
**Age**		
	55.50 ± 9.74	57.29 ± 10.88
**Gender**		
Male	11 (61.1)	16 (76.2)
Female	7 (38.9)	5 (23.8)
**Maximum diameter of tumor (cm)**		
	3.91 ± 1.53	3.51 ± 1.74
**Histology**		
Adenocarcinoma	17 (94.4)	20 (95.2)
Squamous cell carcinoma	1 (5.6)	1 (4.8)
**Smoking status**		
Present or former smoker	10 (55.6)	10 (47.6)
Non-smoker	8 (44.4)	11 (52.4)
**Lymph node status**		
N_0_	11 (61.1)	12 (57.1)
N_1_	3 (16.7)	1 (4.8)
N_2_	4 (22.2)	8 (38.1)
**Tumor differentiation**		
Well	9 (50.0)	10 (47.6)
Moderate or Poor	9 (50.0)	11 (52.4)
**pTMN stage**		
I B high-risk patients ^a^	8 (44.4)	9 (42.9)
Ⅱ	5 (27.8)	5 (23.8)
ⅢA	5 (27.8)	7 (33.3)
***EGFR* mutations**		
19delete	7(38.9%)	9(42.9%)
21 L858R	10(55.6%)	12(57.1%)
Other	1(5.6%)	0

^a^ High-risk patients were defined as patients with poorly differentiated tumors (including lung neuroendocrine tumors, but excluding well-differentiated neuroendocrine tumors), vascular invasion, wedge resection, tumor size > 4 cm, visceral pleural involvement or incomplete lymph node sampling.

### Treatment-related side effects

Four patients in each group had at least one chemotherapy-related adverse event (19% of the chemotherapy plus icotinib treatment group vs. 22% of the chemotherapy-only group). Table 2 summarizes the chemotherapy-related side effects. The most frequent chemotherapy-related complications involved the gastrointestinal tract and marrow suppression during the treatment. These side effects were relatively mild and were mainly assigned grades of 0 or 1 after assessment, with a small number receiving a grade of 2; while no grade 3 side effects or occurrences of intolerable toxicity were observed. No significant differences were identified in the rates of chemotherapy-related adverse events occurring between the two treatment groups.

**Table 2:** Adverse events associated with treatment.

	Chemotherapy		Chemotherapy plus icotinib	
	(n = 18)		(n = 21)	
	n (%)		n (%)	
	Grade 1	Grade 2	Grade 1	Grade 2
Gastrointestinal reactions	3 (16.7%)	1 (5.6%)	3 (14.3%)	0
Marrow suppression	3 (16.7%)	0 (0.0%)	4 (19.0%)	0
Neurotoxicity	3 (16.7%)	1 (5.6%)	4 (19.0%)	0
Liver and kidney damage	2 (11.1%)	1 (5.6%)	4 (19.0%)	0
Allergic reactions	2 (11.1%)	1 (5.6%)	3 (14.3%)	0

In the combined chemotherapy plus icotinib treatment group, three patients presented with grade 1 diarrhea (14.3% in 21), six patients presented with grade 1 rashes on their skin and one case developed a grade 3 rash (33.3% in 21) during the period of icotinib treatment. These side effects were improved after appropriate therapy. Other side effects, including neurotoxicity, liver and kidney damage and allergic reactions, were rarely observed.

### Treatment responses

**Fig 2:**
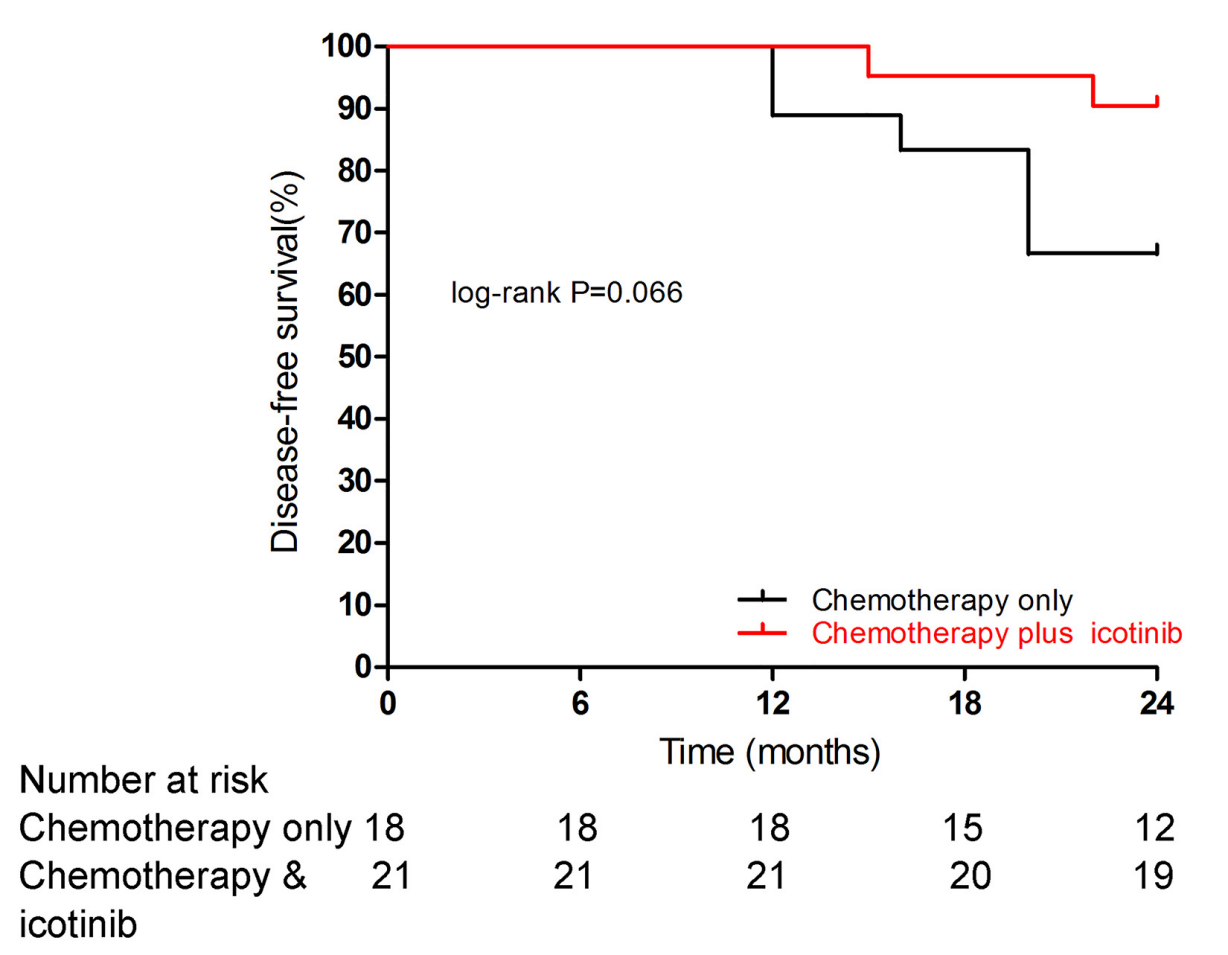
Kaplan-Meier curves for disease-free survival by treatment arm.

All 39 patients recruited for the study have finished the scheduled treatment and were eligible for data analysis. Patient outcomes were followed for up to 24 months after either four cycles of platinum-based doublet chemotherapy (paclitaxel and nedaplatin or lobaplatin) or consolidation therapy (platinum-based doublet chemotherapy supplemented by oral icotinib treatment). At the data cutoff, 6 patients (33.3% in 18) in the chemotherapy group and 2 (9.5% in 21) patients in the chemotherapy plus icotinib group had recurrence or metastasis. The DFS was 21 (100%) in the chemotherapy plus icotinib group vs. 16 (88.9%) in the chemotherapy only group at 12 months (p = 0. 122), 20 (95.2%) vs. 15 (83.3%) at 18 months (p = 0. 225) and 19 (90.5%) vs. 12 (66.7%) at 24 months (p = 0. 066). Fig 2 shows the 24-month Kaplan-Meier curves for both treatment groups. A longer follow-up study is needed to assess the long-term treatment responses of these 39 patients.

### Exploratory subgroup analysis

**Fig 3:**
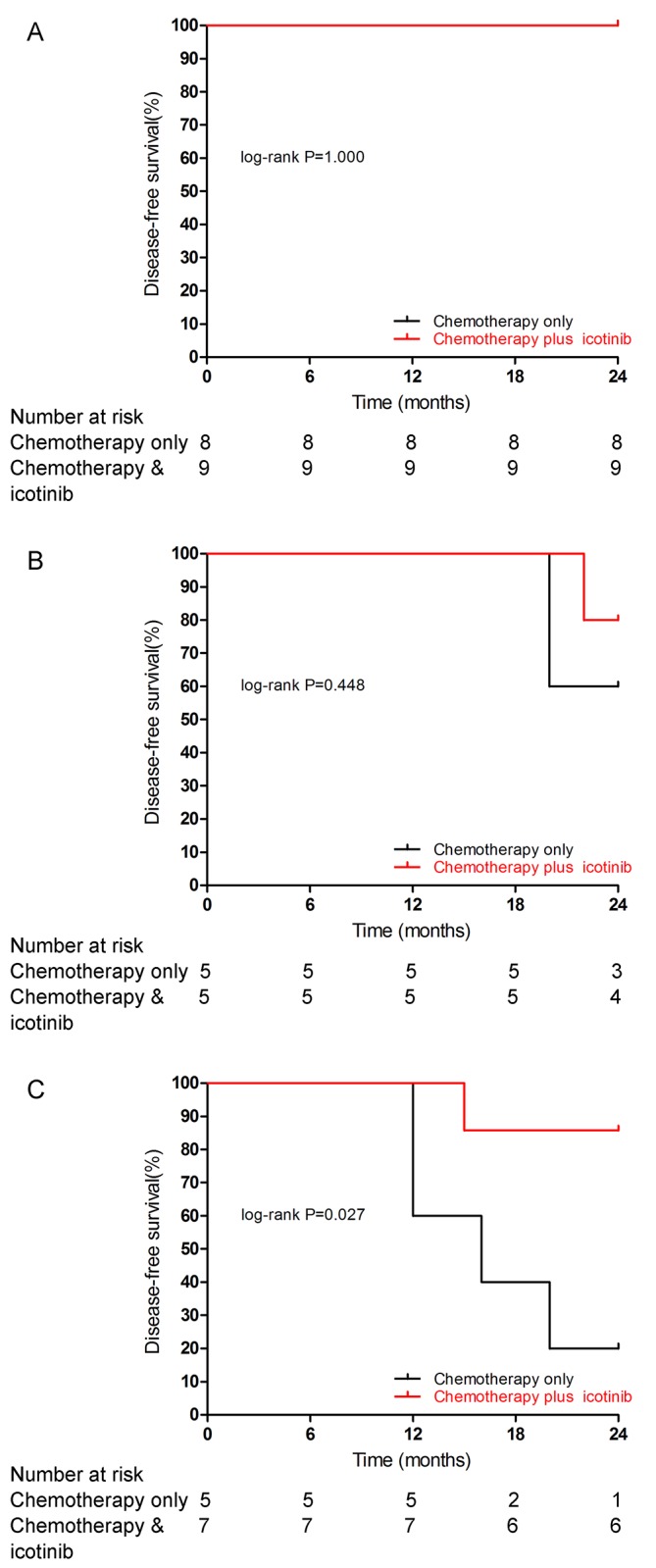
Kaplan-Meier curves for disease-free survival in subgroups. Kaplan-Meier curves for disease-free survival by treatment arm are shown for patients with stage IB (A), Ⅱ (B) or ⅢA (C) NSCLC.

We performed a subgroup analysis of DFS according to the pTNM stage, although this analysis included only 17 patients in the high-risk stage IB subgroup, 10 in the stageⅡsubgroup and 12 in the stage ⅢA subgroup. There was no recurrence event in the high-risk stage IB subgroup during the follow-up period (Fig 3A). The DFS rate was 4 (80.0%) vs 3 (60.0%) in the stageⅡsubgroup (p = 0. 448; Fig 3B) and 6 (85.7%) and 1 (20.0%) in the stage ⅢA subgroup (p = 0. 027; Fig 3C).

## Discussion

This study is a randomized, controlled trial to prospectively compare the icotinib consolidation therapy and adjuvant chemotherapy in completely resected NSCLC with *EGFR* activating mutations. The icotinib treatment was non-inferior to gefitinib in *EGFR*-mutated advanced or metastatic NSCLC and had less drug-related adverse event than gefitinib according to the ICOGEN study [33].

In our study, the patients who received oral icotinib tolerated the drug well and no dose reductions or dose interruptions were necessary during this trial. We observed only one grade 3 skin rash, and the incidence of icotinib-related adverse events was 47.6% in 21 patients. The rate of side effects in our study was notably better than the rate reported in the ICOGEN study, where the incidence of drug-related adverse events in icotinib-treated patients was 61% [33].

With the respect of treatment responses, we found an excellent DFS rate in patients who received combined chemotherapy and oral icotinib treatment (100% DFS after 12 months, 95.2% DFS after 18 months and 90.5% DFS after 24 months). Our results suggested that combined chemotherapy and icotinib treatment tended to elicit a longer duration of DFS when compared with chemotherapy treatment alone in NSCLC patients with *EGFR* sensitive mutations, although the difference in DFS between the two treatment groups did not reach statistical significance (p = 0. 066 at the best) in the log-rank test.

The results of our study are consistent with the results of some other studies in which patients underwent radical resection of NSCLC and were selected to receive adjuvant TKI according to the *EGFR*-mutation status. For example, a retrospective study of 167 patients with *EGFR* sensitive mutations (70% stage IB, 15% stageⅡ, and 15% stage Ⅲ) from the Memorial Sloan-Kettering Cancer Center (New York, USA) showed that adjuvant treatment with *EGFR*-TKIs (gefitinib or erlotinib) could prolong two-year DFS when compared to treatment with platinum-based chemotherapy alone (89% vs. 72%, p = 0.06) [36]. In another example, the phrase 2 single-arm study (SELECT) prospectively demonstrated a two-year DFS rate of 90% in patients with post-surgery stage IA-ⅢA *EGFR*-mutated NSCLC receiving adjuvant erlotinib treatment for 2 years after standard chemotherapy [37]. Now there is a general agreement that activating mutation of *EGFR* is a strong predictor of efficacy for TKI in advanced NSCLC, while the application of adjuvant TKI in NSCLC patients after radical operation is still under research.

To our knowledge, patients with wild-type NSCLC were unlikely to benefit from TKI treatment. Therefore, adjuvant TKI might show no beneficial effect on DFS or OS when patients were not selected according to the *EGFR*-mutation status. In the placebo-controlled BR.19 study, adjuvant gefitinib showed no beneficial effect on DFS (HR, 1.22) or OS (HR, 1.24) for the overall population, neither on DFS (HR, 1.84) or OS (HR, 3.16) for patients with *EGFR*-mutated tumors [38]. It is possible because that the number of patients with *EGFR* mutation-positive tumors was low in the study (only 15 in 503, seven on gefitinib and eight on placebo), the sample size was not enough for a sufficiently effective analysis, and the result of subgroup analysis seems to be underpowered. Another randomized, phase 3 trial (RADIANT) [39] indicated that adjuvant erlotinib did not prolong DFS in the unselected population, while the median DFS duration in the erlotinib treatment group was better than that of chemotherapy-only patients (46.4 vs. 28.5 months, p = 0.0391, not statistically significant according to the hierarchical testing) in a subset of *EGFR*-mutated NSCLC patients.

Our current study suggested improved disease control in selected NSCLC patients. We believe that the *EGFR*-mutation status should be determined before the initial treatment or clinical trial with *EGFR*-TKIs in NSCLC patients. Because all the patients included in our study had resectable tumor lesions, we hypothesized that icotinib therapy might have a synergistic effect to inhibit the circulating tumor cell (CTC) or tiny metastases lesions when combined with standard chemotherapy treatment.

In the subgroup of patients with NSCLC staging ⅢA, the DFS significantly favored chemotherapy plus icotinib (85.7% vs 20.0%, p = 0. 027), while this was not the case in the other subgroups in our study. It is not an isolated result that the survival benefit from adjuvant TKI seems not so obvious in patients with early-stage *EGFR*-mutated NSCLC. In the SELECT study, surprisingly, the two-year DFS in stageⅡ subgroup (73%) was worse than that in stage Ⅲ (92%) [37]. It may be that the *EGFR* pathway plays a less important role in early disease and tumors are not as dependent on this pathway as an oncogenic driver as later disease states, or there exist some interaction between *EGFR* and other signal pathway. A study by researchers at the Ohio State University Comprehensive Cancer Center demonstrated that treatment of *EGFR*-mutated lung cancer cell lines with erlotinib, while showing robust cell death, enriches the ALDH^+^ cells through *EGFR*-dependent activation of the *Notch* pathway [40]. ALDH positivity has been found to be a good marker for a tumor cell subset with stem-like cell properties in lung cancer [41]. This might explain the worsened survival observed in some studies of TKI treatment in early-stage disease. Nevertheless, the biochemical basis of the *EGFR* and *Notch* interaction has been unclear, and likewise its role in lung cancer biology. The specific mechanism still needs further research, while researchers should pay attention to this phenomenon when designing further clinical trials focused on adjuvant TKI treatment.

This clinical trial has some limitations that should be taken into consideration. First, the sample size of 39 patients and 24-month follow-up period may have been too limited. Since the subjects were admitted in a single center, the process of enrollment was not so smooth. A larger sample size and a longer follow-up study would likely have shown more precise differentiation between the DFS rates of icotinib-treated and chemotherapy-only patients. Second, increasing the duration of icotinib treatment or giving icotinib treatment and chemotherapy simultaneously may have resulted in better synergistic control of NSCLC. We would think the preliminary result here was promising, and further studies with larger patient populations recruited from multiple centers might establish the clinical efficiency of adjuvant TKI in selected patients. We look forward to the results of the two ongoing randomized controlled trials focused on adjuvant TKI versus chemotherapy in patients with stageⅡ~ⅢA *EGFR*-mutated NSCLC, the Chinese CTONG1104 study and the Japanese IMPACT study.

## Supporting Information

S1 CONSORT Checklist CONSORT Checklist. (DOC)

S1 Protocol Trial protocol in English. (DOC)

S2 Protocol Trial protocol in Chinese. (PDF)

S1 Table Relevant data underlying the findings described in the manuscript. (XLSX)
