# Supplementary material for: Randomized Adjuvant Chemotherapy of EGFR-Mutated Non-Small Cell Lung Cancer Patients with or without Icotinib Consolidation Therapy
Source: PLoS One. 2015 Oct 16;10(10):e0140794. doi: 10.1371/journal.pone.0140794 (PMC4608803; doi:10.1371/journal.pone.0140794)
Supplement: S1 Protocol — (DOC) [file pone.0140794.s002.doc]

­­**Icotinib as Maintenance Treatment after Chemotherapy for Patients Undergoing Resection of EGFR Mutation-positive Non-Small Cell Lung Cancer**

Principle investigator: Kaican Cai

Institution: Department of Thoracic Surgery, Nanfang Hospital, Southern Medical University

Version: 1.0 Date: 2011.01.20

1. Brief introduction
   1. Purpose of this trial

This trial aims to observe and compare the efficacy and safety of chemotherapy or chemotherapy plus icotinib in patients undergoing resection of EGFR mutation-positive non-small cell lung cancer stagingⅠB (with high risk factor) to ⅢA.

- 1. Backgrounds

Lung cancer currently is the leading cause of cancer-related mortality worldwide, of which non-small cell lung cancer (NSCLC) accounts for more than 85% ^[1]^. NSCLC staging I, Ⅱ and part of ⅢA (T_3_N_1-2_M_0_; T_1-2_N_2_M_0_; T_4_N_0-1_M_0_) are possible to be completely resected. Based on the basic and clinical researches in decade years, it is confirmed that the postoperative adjuvant chemotherapy could make survival benefit in the NSCLC patients with stage IB (with high risk factors) to stage ⅢA. The survival benefit from chemotherapy, however, is still limited ^[2-5]^.

Along with the development of molecular biology and relevant clinical researches, molecularly targeted therapy has attracted great attention and is considered as the most promising strategy for lung cancer therapy in the 21st century. Epidermal growth factor receptor tyrosine kinase inhibitor (EGFR-TKI) is one of the most exploited areas.

EGFR-TKIs can competitively inhibit the ATPs binding to the intracellular areas of EGFR, thus inhibiting its signaling pathway and achieving the anti-tumor effect. EGFR-TKIs are considered as the promising therapeutic drugs for the effectiveness in the treatment of EGFR-mutated NSCLC because patients with EGFR activating mutations are notably sensitive to EGFR-TKIs ^[6]^. There are four drug-sensitive mutations, including point mutations in exon 18 (G719A/C), 21 (L858R and L861Q) and in-frame deletions in exon 19. These sensitizing EGFR mutations are found in approximately 10% of Caucasian patients with NSCLC and up to 50% of Asian patients. Thus far, three kinase domain mutations are associated with drug resistance: an exon 19 point mutation (D761Y), an exon 20 point mutation (T790M) and an exon 20 insertion ^[7]^.

Several clinical trials, such as IPASS ^[8]^, WJTOG3405 ^[9]^ and NEJ002 ^[10]^, have demonstrated that a significant survival benefit is associated with EGFR-TKIs treatment in patients with EGFR-mutanted advanced NSCLC. Different combined models of chemotherapy and EGFR-TKIs used in advanced NSCLC are also under research. It is confirmed that TKIs as maintenance treatment after chemotherapy significantly prolong PFS in advanced non-small cell lung cancer. The WJTOG0203 trial enrolled 604 patients with advanced stage (IIIB/IV) NSCLC to evaluate whether gefitinib improves survival as sequential therapy after platinum-doublet chemotherapy. There was a statistically significant improvement in PFS in the chemotherapy plus gefitinib group (4.60 vs 4.27months, HR=0.68, 95% CI: 0.57 to 0.80, P＜0.001); however, OS results did not reach statistical significance (13.7 vs 12.9months, HR=0.86, 95%CI: 0.72-1.03, P=0.10) ^[11]^. The phase Ⅲ, placebo-controlled sequential erlotinib in unresectable NSCLC (SATURN) study assessed the use of erlotinib as maintenance therapy in patients with non-progressive disease following first-line platinum-doublet chemotherapy. 884 patients were analysable for PFS; 437 in the erlotinib group and 447 in the placebo group. Median PFS was significantly longer with erlotinib than with placebo: 12.3 weeks for patients in the erlotinib group versus 11.1 weeks for those in the placebo group (HR=0.71, 95% CI: 0.62-0.82, P＜0.0001). PFS was also significantly longer in patients with EGFR-positive immunohistochemistry who were treated with erlotinib (n=307) compared with EGFR-positive patients given placebo (n=311, median PFS 12.3 vs 11.1 weeks, HR=0.69, 95% CI: 0.58-0.82, P＜0.0001) ^[12]^.

This study aims to investigate the application of EGFR-TKIs as adjuvant therapy in NSCLC patients after radical operation. We plan to assess the efficacy and safety of chemotherapy with or without icotinib in patients undergoing the resection of stage IB to ⅢA EGFR-mutated NSCLC.

- 1. Possible damage to participants in this trial
     1. Chemotherapy-related adverse events:

The digestive system: decreased appetite, nausea and vomiting, stomachache, diarrhea, peptic ulcer, perforation, pancreatic function injury, liver and kidney damage, etc.

The hematopoietic system: marrow suppression, etc.

The cardiovascular system: myocardial injury, arrhythmia, thrombosis or embolism, shock, etc.

The respiratory system: interstitial damage, etc.

The urinary system: kidney damage, etc.

The reproductive system: hypogonadism, reproductive suppression, teratogenic, etc.

The nervous system: neurotoxicity, etc.

The locomotor system: muscle soreness, etc.

Others: baldness, skin rashes, dermatitis, etc.

- - 1. TKI-related adverse events

Diarrhea, etc.

Oral lesions: pain, erythema, ulcer, etc.

Skin: pruritus, dry dermatitis, erythema, papula, dermatitis, peeling, etc.

- - 1. Others: The rest of the risks are the same with accepted surgery, such as lung surgery and closed thoracic drainage.
  1. Possible benefit to participants in this trial

Treatment in this trial may prolong DFS, delay disease progression and improve the quality of life of patients, but these benefits may not be observed in every participant.

1. Study Design

Type of design: a prospective randomized controlled trial

Random method: a random digits table

Masking: Open Label

Enrollment: 58 [Anticipated]

Anticipated start and competition time: Jan 2011 to Jan 2018

- 1. Patients
     1. Inclusion Criteria:

Over 18 years of age

Patients undergoing completely resection of EGFR mutation-positive NSCLC

Staging ⅠB (with high risk factor) to ⅢA

PS = 0 or 1

Adequate hematological, biochemical and organ functions

- - 1. Exclusion Criteria:

Any EGFR-TKI resistant mutations

Systemic anticancer therapy prior to surgery

Other malignancies before or during the study

Any unstable illness

Pregnancy or lactation

- 1. Treatment

Eligible patients were randomly assigned in a 1:1 ratio to the chemotherapy-only or chemotherapy plus icotinib treatment group. All patients received four cycles of platinum-based doublet chemotherapy (150 mg/m^2^ paclitaxel plus 80 mg/m^2^ nedaplatin or 30mg/m^2^ lobaplatin on day one of a three-week cycle). Two weeks after chemotherapy completed, patients assigned to the consolidation therapy group began oral icotinib treatment (125 mg, thrice daily). Icotinib treatment continued for four to eight months, or until the occurrence of disease relapse, metastasis or unacceptable icotinib or chemotherapy toxicity.

- 1. Drugs in this trial
     1. TKI

Common name: Icotinib Hydrochloride Tablets

Trade name: Conmana (Zhejiang betta pharmaceutical Co., LTD, China)

21 pills per package

This product is a brownish red film-coated tablet, and shows white without coating.

- - 1. Chemotherapy drugs

The chemotherapy regimen is 150 mg/m^2^ paclitaxel plus 80 mg/m^2^ nedaplatin or 30mg/m^2^ lobaplatin on day one of a three-week cycle

- - 1. Others

Other drugs such as omeprazole, tropisetron and recombinant human granulocyte colony-stimulating factor may be used to deal with the possible chemotherapy-related adverse events.

- 1. Supplementary examinations in this trial

Clinical stage before surgery and treatment response of adjuvant therapy will be assessed by: 1) Positron emission tomography-computed tomography (PET-CT) or 2) X-ray computed tomography (CT) scan, magnetic resonance imaging (MRI), bone scan and abdominal ultrasound, whether a bronchoscopy need to take was depended. Other possible examinations includes chest X-ray radiography, electrocardiogram, pulmonary function tests, and so on.

1. Endpoints and follow-up
   1. The primary endpoint

Disease free survival (DFS): the time from surgery to the first confirmed occurrence of disease relapse or metastasis

- 1. The secondary endpoint

The toxicity of chemotherapy and oral icotinib treatment: according to the World Health Organization Toxicity Grading Scale for Determining the Severity of Adverse Events

- 1. Follow-up

Treatment response was assessed by: 1) Positron emission tomography-computed tomography (PET-CT) or 2) X-ray computed tomography (CT) scan, magnetic resonance imaging (MRI), bone scan and abdominal ultrasound at the beginning of the fourth chemotherapy cycle and every six months thereafter.

1. Data Management and Statistic Analysis
   1. Data Management

Patients will get a specific coding number according their sequence of enrollment. Their information and test data will be recorded in the case report form (CRF) by the researchers. The CRFs will be stored specifically and only be checked by the researchers.

- 1. Statistic Analysis

The intention-to-treat population comprised all randomly assigned patients. The per-protocol population comprised patients who finished the planned treatment program. Kaplan-Meier curves were used to describe survival data, and a two-sided log-rank test was used to compare the two treatment groups. The Cox proportional hazards model was used to analyze DFS data with covariates. Clinical measurements were analyzed with the Student’s *t*-test, unordered enumeration data with the χ^2^ test and ranked data with the Wilcoxon rank sum test. A *p* ≤ 0.05 was considered statistically significant. All statistical analyses were performed using SPSS 13.0 statistical software (SPSS Inc., Chicago, IL, USA).

1. Adverse events
   1. Recorded contents about adverse events

The recorded contents about adverse events includes the happening time and the duration, the description of the adverse events and all related symptoms, the severity, examinations and treatments to deal with the adverse events and the results of therapy. Whether adverse events are related to this trial should also be recorded.

- 1. Severe adverse event reports

When severe adverse events happened, the researchers should report to the pharmaceutical supervisory and administrative department within 24 hours, and report to the sponsor immediately and the ethics committee as soon as possible. Researchers should record the severe adverse events are reported on what time, in what way (such as telephone, fax or written) and to whom.

- 1. Treatment measures to deal with severe adverse events

When severe adverse events happened, the most important issue is to save lives. Researchers should take active measures to rescue, look for the causes and deal with it.

- 1. Follow-up with an adverse event

For patients who suffered from adverse events, researchers should follow closely about the outcomes of the adverse events and whether it happens again.

1. The ethical principles

This trial must perform in accordance with the Declaration of Helsinki. All patients should provide written informed consent before participating in this study.

1. The quality control

The researchers should strictly abide by the research protocol and regulations related to clinical trials. The participants should receive medical care in this protocol, but not receive other anticancer therapy during the study.

**References:**

[1] Network N C C, Others. NCCN clinical practice guidelines in oncology: non-small cell lung cancer[J]. Fort Washington, PA: NCCN. 2010 version 2.

[2] Winton T, Livingston R, Johnson D, et al. Vinorelbine plus cisplatin vs. observation in resected non-small-cell lung cancer[J]. N Engl J Med. 2005, 352(25): 2589-2597.

[3] Douillard J Y, Rosell R, De Lena M, et al. Adjuvant vinorelbine plus cisplatin versus observation in patients with completely resected stage IB-IIIA non-small-cell lung cancer (Adjuvant Navelbine International Trialist Association [ANITA]): a randomised controlled trial[J]. Lancet Oncol. 2006, 7(9): 719-727.

[4] Roselli M, Mariotti S, Ferroni P, et al. Postsurgical chemotherapy in stage IB nonsmall cell lung cancer: Long-term survival in a randomized study[J]. Int J Cancer. 2006, 119(4): 955-960.

[5] Wang S, Rong T, Ou W, et al. [A prospective randomized study of adjuvant chemotherapy in completely resected stage IIIA-N2 non-small cell lung cancer][J]. Zhongguo Fei Ai Za Zhi. 2006, 9(5): 434-438.

[6] Sirotnak F M, Zakowski M F, Miller V A, et al. Efficacy of cytotoxic agents against human tumor xenografts is markedly enhanced by coadministration of ZD1839 (Iressa), an inhibitor of EGFR tyrosine kinase[J]. Clin Cancer Res. 2000, 6(12): 4885-4892.

[7] Riely G J, Politi K A, Miller V A, et al. Update on epidermal growth factor receptor mutations in non-small cell lung cancer[J]. Clin Cancer Res. 2006, 12(24): 7232-7241.

[8] Mok T S, Wu Y L, Thongprasert S, et al. Gefitinib or carboplatin-paclitaxel in pulmonary adenocarcinoma[J]. N Engl J Med. 2009, 361(10): 947-957.

[9] Mitsudomi T, Morita S, Yatabe Y, et al. Gefitinib versus cisplatin plus docetaxel in patients with non-small-cell lung cancer harbouring mutations of the epidermal growth factor receptor (WJTOG3405): an open label, randomised phase 3 trial[J]. Lancet Oncol. 2010, 11(2): 121-128.

[10] Maemondo M, Inoue A, Kobayashi K, et al. Gefitinib or chemotherapy for non-small-cell lung cancer with mutated EGFR[J]. N Engl J Med. 2010, 362(25): 2380-2388.

[11] Takeda K, Hida T, Sato T, et al. Randomized Phase III Trial of Platinum-Doublet Chemotherapy Followed by Gefitinib Compared With Continued Platinum-Doublet Chemotherapy in Japanese Patients With Advanced Non-Small-Cell Lung Cancer: Results of a West Japan Thoracic Oncology Group Trial (WJTOG0203)[J]. Journal of Clinical Oncology. 2010, 28(5): 753-760.

[12] Cappuzzo F, Ciuleanu T, Stelmakh L, et al. Erlotinib as maintenance treatment in advanced non-small-cell lung cancer: a multicentre, randomised, placebo-controlled phase 3 study[J]. The Lancet Oncology. 2010, 11(6): 521-529.
