# Supplementary material for: Randomized Adjuvant Chemotherapy of EGFR-Mutated Non-Small Cell Lung Cancer Patients with or without Icotinib Consolidation Therapy
Source: PLoS One. 2015 Oct 16;10(10):e0140794. doi: 10.1371/journal.pone.0140794 (PMC4608803; doi:10.1371/journal.pone.0140794)
Supplement: S2 Protocol — (PDF) [file pone.0140794.s003.pdf]

版本号：1.0　　　日期：2011.01.20

化疗+埃克替尼维持治疗对EGFR突变型非小细胞肺癌根治术后患者生存获益的影响

主要研究者：蔡开灿

研究单位：南方医科大学南方医院　　胸心血管外科

保密声明：

本研究方案中所包含的信息仅提供给本项目的研究者、伦理委员会和相关机构审阅。在未得到主要研究者（PI）批准的情况下，严禁将任何信息告知与本研究无关的第三方。

一、 试验目的、背景及已知对人体的可能危险与受益

1. **试验目的：**观察并比较高危 IB-IIIA 期非小细胞肺癌根治术后 EGFR 突变型患者接受化疗或化疗+埃克替尼维持治疗的疗效与安全性
2. **背景资料：**

肺癌是世界范围内肿瘤死亡的首要原因，其中非小细胞肺癌（non-small cell lung cancer，NSCLC）占所有肺癌病例的 85%以上[1]。目前在肺癌的外科治疗中，Ⅰ、Ⅱ期和部分ⅢA 期（$T_3N_{1-2}M_0$；$T_{1-2}N_2M_0$；$T_4N_{0-1}M_0$）NSCLC 患者可行完全性切除手术。NSCLC 完全性切除术后的辅助化疗在一定程度上提高了患者的 5 年生存率。术后辅助化疗的相关共识如下：Ⅱ~ⅢA 期 NSCLC 患者推荐术后行辅助化疗（1A 类推荐）；IA 期 NSCLC 患者推荐术后不行辅助化疗（1A 类推荐）；切缘阴性的 IB 期高危人群需行辅助化疗（2B 类推荐），高危因素可能包括低分化肿瘤（包括肺神经内分泌肿瘤、但除外高分化神经内分泌肿瘤）、脉管受侵、楔形切除、肿瘤直径＞4cm、脏层胸膜受侵和不完全的淋巴结取样（N_x），这些独立因素可能不是适应症，但在决定辅助化疗时应该考虑。但术后辅助化疗对患者的生存率提高仍较为有限[2-5]。

近年来，随着肿瘤细胞信号传导通路的研究不断深入，人们越来越清晰地认识到肿瘤的发生、发展、复发及转移的分子机制，针对肿瘤细胞特异性分子靶点的分子靶向治疗逐渐成为研究的热点。其中对表皮生长因子受体（epidermal growth factor receptor, EGFR）及其分子靶向药物的研究最为深入。

表皮生长因子受体酪氨酸激酶抑制剂(epidermal growth factor receptor tyrosine kinase inhibitors，EGFR-TKIs)如吉非替尼、厄罗替尼及埃克替尼通过竞争性地抑制 ATP 与 EGFR 胞内区结合，抑制酪氨酸激酶活化、阻断下游信号传导通路，从而阻断细胞周期、促进肿瘤细胞凋亡、阻止肿瘤生长及转移等，达到抗肿瘤治疗的目的。EGFR 敏感突变导致其酪氨酸激酶区构相的改变，使 TKI 药物更易与 EGFR 结合，增加了 TKI 药物的敏感性[6]。目前已有四类突变状态被认为可增加 TKI 药物的敏感性，分别是 19 外显子缺失（19Del）、21 外显子突变（21 L858R）、21 外显子点突变（21 L861Q）、18 外显子点突变（18 G719A/C）；另有三类突变状态被证实与 TKI 耐药相关，分别是 19 外显子点突变（19 D761Y）、20 外显子点突变（20 T790M）、20 外显子插入[7]。

EGFR-TKIs 用于治疗 NSCLC 的临床研究首先在晚期患者中取得突破。IPASS[8]、WJTOG3405[9]、NEJ002[10]等研究证实：较之传统化疗，EGFR-TKIs 一线治疗 EGFR 敏感突变的晚期 NSCLC 患者可提高 PFS 和改善生活质量。并有研究者开始探究化疗与 EGFR-TKIs 的不同组合模式在晚期 NSCLC 患者中的应用。其中化疗后 TKI 维持治疗这一模式在晚期患者中获得了较好的生存获益。日本的 WJTOG0203 研究纳入 604 例未经过 EGFR 基因突变检测的ⅢB～Ⅳ期 NSCLC 患者，随机接受 6 周期含铂两药化疗，或 3 周期化疗后口服吉非替尼维持治疗；结果显示维持治疗组 PFS 显著延长（4.60 vs 4.27 个月，HR=0.68，95%CI：0.57-0.80，P＜0.001），但两组 OS 无显著差异（13.7 vs 12.9 月，HR=0.86，95%CI：0.72-1.03，P=0.10）[11]。SATURN 是一项多中心Ⅲ期临床随机试验，共纳入 1949 例晚期 NSCLC 患者，在四周期含铂两药化疗后评估病情变化，其中 889 例病情未进展的患者继续参与研究，随机分组接受口服厄洛替尼（150mg/天）或安慰剂对照，至病情进展后停药。结果显示，厄洛替尼组的 PFS 较安慰剂组显著延长（12.3 vs 11.1 周，HR=0.71，95% CI：0.62-0.82，P＜0.0001）；亚组分析提示 EGFR 突变的患者亦从维持治疗中明显获益，PFS 为 12.3 vs 11.1 周（HR=0.69，95% CI：0.58-0.82，P＜0.0001）[12]。

本研究尝试将化疗+埃克替尼维持治疗这一方案引入 EGFR 突变型 NSCLC 根治性术后患者的辅助治疗中，观察其疗效及安全性。

3. 已知对人体的可能危险：

（1）化疗相关并发症：

消化系统（食欲减退、恶心、呕吐；腹痛、腹泻、消化道溃疡、穿孔、胰腺功能损伤等）；

造血系统（骨髓抑制等）；

心血管系统（心肌损伤、心律失常，血栓形成或栓塞，休克等）；

肝脏毒性（肝功能损伤、肝功能衰竭等）；

呼吸系统（肺间质损害等）；

泌尿系统：（肾功能受损等）；

生殖系统：（性功能减退、生殖抑制，致畸等）；

中枢、神经系统（头晕、头痛；神经损伤等）；

运动系统（肌肉酸痛等）；

其它如脱发、皮疹、皮炎等。

（2）靶向药物相关毒性反应：

腹泻；

口腔病变（疼痛、红斑、溃疡）；

皮肤（红斑、丘疹、干性皮炎、瘙痒、皮炎性脱皮）等。

（3）其余风险与所行手术（如肺部手术、胸腔闭式引流术）等相同。

4. **可能的受益：**本研究可能会延长根治术后患者的无疾病生存期（DFS），延缓疾病进展，改善患者生活质量；可能没有受益。

二、 试验设计类型及随机化分组方法及设盲水平

1. **试验设计类型：**前瞻性随机对照试验
2. **分组方法：**随机分组（随机数字表法）
3. **设盲水平：**不设盲

三、 受试者选择

患者入院后完善相关辅助检查后，按如下标准进行筛选或剔除

1. **受试者入选标准：**（1）已接受解剖性肺叶切除、系统性肺门和纵隔淋巴结清扫术的NSCLC 患者，术中完全切除病灶，术后病理分期为高危 IB 期～ⅢA 期患者；

（2）EGFR 突变检测发现一种或多种敏感突变；

（3）年龄≥18 岁；

（4）血液学指标、肝肾功能在正常范围；

（5）体力状态（performances status, PS）评分为 0 或 1 分；

（6）入组前未接受过化疗或放疗等任何抗肿瘤治疗；

（7）入组前或入组时未罹患其他类型恶性肿瘤；

（8）知情并同意术后辅助治疗。

2. **受试者排除标准：**（1）检出任一类 EGFR 耐药突变；

（2）罹患其他疾病且病情尚未稳定；

（3）妊娠期或哺乳期妇女。

3. 受试者剔除标准：1. 研究期间患者决定退出者

2. 研究期间违背研究方案者

3.研究期间患者因药物副作用或其他原因不宜继续接受该治疗方案

4. 预计试验所需受试者总例数：58 例

四、 研究用药

1. 药物名称，剂型

研究用药为盐酸埃克替尼片（凯美纳，浙江贝达药业股份有限公司），本品为棕红色薄膜衣片，去除包衣后显类白色。

2. 分组及用药方法：

入组患者按 1:1 随机分配至化疗组或序贯治疗组。化疗组从术后第四周起给予含铂两药化疗，以 21 天为一个周期，化疗共四个周期后定期复查。序贯治疗组接受相同的四周期化疗，第四次化疗结束两周后开始口服盐酸埃克替尼片，持续口服 4-8 个月。

3. 合并用药：

① 化疗药：化疗方案为紫杉醇（150mg/m^2，第 1 天）+铂类（奈达铂 80mg/m^2 或洛铂 30mg/m^2，第 1 天）

②辅助用药：治疗期间需使用护胃药（如奥美拉唑）、止吐药（如托烷司琼）、升白药（如重组人粒细胞集落刺激因子）等。

4. 药物包装和标签：

铝塑铝包装。每板 21 片，每盒装 1 板。

五、 治疗前后所需辅助检查、检验

根据病情需要完善相关辅助检查、检验，包括：胸片、心电图、肺功能、胸部增强 CT、电子纤维支气管镜、骨显像、头颅 MRI、超声、血常规、肝肾功能等。其中血常规、肝肾功能、胸片需行多次检查。

六、 治疗过程

1. 按以下程序进行治疗：

患者均在全麻、双腔气管插管、单肺通气条件下行胸腔镜下单向式肺癌根治术。术后第三周开始接受含铂两药化疗，以 21 天为一周期，共四个周期；维持治疗组在化疗结束两周后开始口服盐酸埃克替尼片，持续用药 4-8 个月。接受药物治疗期间及治疗后需定期复查。

2. 治疗过程中及治疗后可能存在的不良反应：

（1）化疗过程中可能出现骨髓抑制、肝肾功能损害及胃肠道反应（如恶心呕吐等）等化疗副作用。

（2）服用 EGFR-TKIs 期间可能出现副作用：腹泻；口腔病变（疼痛、红斑、溃疡）；皮肤（红斑、丘疹、干性皮炎、瘙痒、皮炎性脱皮）等。

七、 治疗后的临床观察、随访及保证受试者依从性的措施

1. **治疗后的主要观察指标为：**

   患者无疾病生存期：手术后至发现疾病复发或转移的时间；

   化疗后毒性反应：胃肠反应、骨髓抑制、神经毒性、肝肾损害、过敏反应；

   EGFR-TKIs 药物副作用

2. **出院后随访：**第四次化疗开始前、四个周期治疗结束后第三个月行全身检查，之后每半年复查一次，方案为全身 PET-CT（或头颅 MRI+胸部增强 CT+全身骨显像+腹部超声），观察肿瘤有无复发或转移。
3. **保证受试者依从性的措施：**出院医嘱

八、 疗效评定

无疾病生存期：手术后至发现疾病复发或转移的时间，以月为单位进行统计。

八、 受试者编码及病例报告表的保存

1. **受试者编码：**按患者就诊时间顺序编码

2. **病例报告表的保存：**由研究者记录后，存放于指定文件夹内，该文件夹内容只能研究者观看。

九、 不良事件

1. **记录应包括：**不良事件及所有相关症状的描述；不良事件发生的时间及持续时间；不良事件的严重程度；因不良事件所做的检查和治疗；不良事件的最终结果；判断不良事件是否与试验有关。
2. **严重不良事件报告方法：**遇到严重不良事件时，研究者必须在 24 小时之内报告药品监督管理部门，并立即报告申办者和尽快报告伦理委员会。在原始资料中应记录何时、以何种方式（如电话、传真或书面）、向谁报告了严重不良事件。
3. **严重不良事件处理措施：**遇到严重不良事件时，应以抢救患者生命为目标，积极抢救，维持患者生命体征，寻找导致患者出现该症状的原因，并予以处理。
4. **随访：**对出现了不良事件的患者，应于定期随访时询问该不良事件是否好转、消失，是否再度发生。

十、 数据的管理、分析与统计

1. **试验所得数据：**记录于病例报告表，由研究者统一保存、管理。
2. **数据的分析与统计：**

(1)无疾病生存期:手术后至发现疾病复发或转移的时间;统计方法采用 Kaplan-Meier 描述生存曲线，Log-rank 检验显著性，Cox 比例风险模型进行多因素分析；
(2）化疗后毒性反应：胃肠反应、骨髓抑制、神经毒性、肝肾损害、过敏反应；采用非参数检验;
(3）EGFR-TKIs 药物副作用，采用非参数检验。

十一、 质量控制及保证

研究者应严格按照研究方案执行试验操作，并嘱受试者遵守研究方案，不进行研究方案以外的治疗。

十二、 试验预期时间

2011.01-2018.01。

十三、 试验结束后随访和医疗措施

试验结束后随访继续按照研究方案进行，如受试者出现任何不适可随时来咨询研究者。

十四、 试验过程应坚持的伦理学原则

试验过程应遵守《赫尔辛基宣言》相关伦理原则，力求“公正、尊重人格、力求使受试者最大程度受益和尽可能避免伤害”，所有患者接受治疗前均需签署书面知情同意书。

十五、 各方承担的职责

1. 研究者应严格遵守国家关于临床试验的相关规定及研究方案。
2. 受试者应遵守研究方案提出的治疗措施。

References:

[1] Network N C C, Others. NCCN clinical practice guidelines in oncology: non-small cell lung cancer[J]. Fort Washington, PA: NCCN. 2010 version 2.

[2] Winton T, Livingston R, Johnson D, et al. Vinorelbine plus cisplatin vs. observation in resected non-small-cell lung cancer[J]. N Engl J Med. 2005, 352(25): 2589-2597.

[3] Douillard J Y, Rosell R, De Lena M, et al. Adjuvant vinorelbine plus cisplatin versus observation in patients with completely resected stage IB-IIIA non-small-cell lung cancer (Adjuvant Navelbine International Trialist Association [ANITA]): a randomised controlled trial[J]. Lancet Oncol. 2006, 7(9): 719-727.

[4] 王思愚，戎铁华，区伟，等. III A-N2期非小细胞肺癌术后化疗随机对照研究结果[J]. 中国肺癌杂志. 2006，9(5):434-438.

[5] Roselli M, Mariotti S, Ferroni P, et al. Postsurgical chemotherapy in stage IB nonsmall cell lung cancer: Long-term survival in a randomized study[J]. Int J Cancer. 2006, 119(4): 955-960.

[6] Sirotnak F M, Zakowski M F, Miller V A, et al. Efficacy of cytotoxic agents against human tumor xenografts is markedly enhanced by coadministration of ZD1839 (Iressa), an inhibitor of EGFR tyrosine kinase[J]. Clin Cancer Res. 2000, 6(12): 4885-4892.

[7] Riely G J, Politi K A, Miller V A, et al. Update on epidermal growth factor receptor mutations in non-small cell lung cancer[J]. Clin Cancer Res. 2006, 12(24): 7232-7241.

[8] Mok T S, Wu Y L, Thongprasert S, et al. Gefitinib or carboplatin-paclitaxel in pulmonary adenocarcinoma[J]. N Engl J Med. 2009, 361(10): 947-957.

[9] Mitsudomi T, Morita S, Yatabe Y, et al. Gefitinib versus cisplatin plus docetaxel in patients with non-small-cell lung cancer harbouring mutations of the epidermal growth factor receptor (WJTOG3405): an open label, randomised phase 3 trial[J]. Lancet Oncol. 2010, 11(2): 121-128.

[10] Maemondo M, Inoue A, Kobayashi K, et al. Gefitinib or chemotherapy for non-small-cell lung cancer with mutated EGFR[J]. N Engl J Med. 2010, 362(25): 2380-2388.

[11] Takeda K, Hida T, Sato T, et al. Randomized Phase III Trial of Platinum-Doublet Chemotherapy Followed by Gefitinib Compared With Continued Platinum-Doublet Chemotherapy in Japanese Patients With Advanced Non-Small-Cell Lung Cancer: Results of a West Japan Thoracic Oncology Group Trial (WJTOG0203)[J]. Journal of Clinical Oncology. 2010, 28(5): 753-760.

[12] Cappuzzo F, Ciuleanu T, Stelmakh L, et al. Erlotinib as maintenance treatment in advanced non-small-cell lung cancer: a multicentre, randomised, placebo-controlled phase 3 study[J]. The Lancet Oncology. 2010, 11(6): 521-529.

临床试验方案确认签字页

化疗+埃克替尼维持治疗对EGFR突变型非小细胞肺癌根治术后患者生存获益的影响

主要研究者关于方案的同意书：

我已经认真阅读过本方案，我同意方案中包括的所有用来进行研究的必要的信息，并且我同意按方案所描述的内容执行。我明白缺少伦理委员会批准的情况下，试验不得启动，并且要完全遵守本单位的相关规定。

需要获得所有参加的受试者的知情同意书和相应的记录文件。签署知情同意之后，将根据赫尔辛基宣言，以及关于新药和已批准药物和相关试验技术的临床应用的法律法规的要求，开展临床试验。

主要研究者姓名：蔡开灿

研究单位：南方医科大学南方医院胸心血管外科

研究地址：广州市广州大道北 **1838** 号

主要研究者签字： 日期：
